# Recent Advances in Food-Packing, Pharmaceutical and Biomedical Applications of Zein and Zein-Based Materials

**DOI:** 10.3390/ijms151222438

**Published:** 2014-12-04

**Authors:** Elisângela Corradini, Priscila S. Curti, Adriano B. Meniqueti, Alessandro F. Martins, Adley F. Rubira, Edvani Curti Muniz

**Affiliations:** 1 Departmento de Engenharia de Materiais, Universidade Tecnológica Federal do Paraná (UTFPR-LD), Avenida dos Pioneiros, 3131, 86036-370 Londrina-PR, Brazil; E-Mail: ecorradini@utfpr.edu.br; 2 Departmento de Química, Universidade Tecnológica Federal do Paraná (UTFPR-LD), Avenida dos Pioneiros, 3131, 86036-370 Londrina-PR, Brazil; E-Mail: priscilacurti@utfpr.edu.br; 3 Programa de Pós-graduação em Biotecnologia Aplicada à Agricultura, Universidade Paranaense (UNIPAR), 87502-210 Umuarama-PR, Brazil; E-Mail: adrianomeniquetti@hotmail.com; 4 Coordenação do Curso de Agronomia, Universidade Tecnológica Federal do Paraná (UTFPR-DV), Estrada para Boa Esperança, 85660-000 Dois Vizinhos-PR, Brazil; E-Mail: afmartins50@yahoo.com.br; 5 Departamento de Química, Universidade Estadual de Maringá (UEM), Av. Colombo, 5790, 87020-900 Maringá-PR, Brazil; E-Mail: afrubira@uem.br

**Keywords:** zein, zein-based materials, biomedical application, drug delivery, zein hydrolysates applied for reducing blood pressure, colloidal particles of zein, food-packing

## Abstract

Zein is a biodegradable and biocompatible material extracted from renewable resources; it comprises almost 80% of the whole protein content in corn. This review highlights and describes some zein and zein-based materials, focusing on biomedical applications. It was demonstrated in this review that the biodegradation and biocompatibility of zein are key parameters for its uses in the food-packing, biomedical and pharmaceutical fields. Furthermore, it was pointed out that the presence of hydrophilic-hydrophobic groups in zein chains is a very important aspect for obtaining material with different hydrophobicities by mixing with other moieties (polymeric or not), but also for obtaining derivatives with different properties. The physical and chemical characteristics and special structure (at the molecular, nano and micro scales) make zein molecules inherently superior to many other polymers from natural sources and synthetic ones. The film-forming property of zein and zein-based materials is important for several applications. The good electrospinnability of zein is important for producing zein and zein-based nanofibers for applications in tissue engineering and drug delivery. The use of zein’s hydrolysate peptides for reducing blood pressure is another important issue related to the application of derivatives of zein in the biomedical field. It is pointed out that the biodegradability and biocompatibility of zein and other inherent properties associated with zein’s structure allow a myriad of applications of such materials with great potential in the near future.

## 1. Introduction

Zein is the main form of protein storage contained in the endosperm tissue of corn and comprises almost 80% of the whole protein content in the corn [1]. In the past, zein was considered more of a by-product of corn processing industries; the consensus indicated zein to be a low-valued material without important potential technological uses [1,2,3]. However, due to several recent methodologies and developing processes allowing applications in different fields, nowadays, there is new thought related to zein and zein-based materials towards considering them as more valuable materials [4]. Potential applications of zein include uses as biodegradable plastics, fibers, adhesives, coatings, ceramics, inks, cosmetics, textiles and chewing gum [2]. The medical and pharmaceutical fields are very two important fields in which materials based on zein can also be applied. For instance, the fabrication of mats using electrospinning for cell culture substrates applied to tissue engineering appears realistic and not just a promise [5,6,7,8]. However, currently, the most successful applications of zein-based biodegradable materials in the pharmaceutical and food industries are as fibers and formulations to act as coating agents [9,10]. The good mechanical properties of plasticized-zein or of zein blended with other moieties and also the film-forming ability of zein and zein-containing materials are, among others, very important characteristics. However, the biodegradability and biocompatibility are key parameters that enable new uses of zein and zein-based materials in biotechnological areas [5].

Even though some reviews focusing on zein and zein-based products have been published [5,11], new publications show new methodologies to prepare, characterize and apply materials based on zein, plasticized-zein, zein-derivatives (through chemically modifying processes, such as hydrolysis, cross-linking,* etc.*) or blending zein in a physical mixture with other polymers (synthetic or natural). A quick search made by the authors in the ISI (Web of Science^©^) database using the word “zein” revealed more than 2000 papers (*ca*. 80% of them being published in the last two decades) illustrating the importance of zein and zein-based materials in both academic and technological fields.

In this review, the authors attempt to highlight and describe some zein and zein-based materials, updating information concerning the basic structure, properties, changes to the properties (by chemical modification, blending, mixing), colloidal particle formation, degradation (enzymatic or not) and applications of zein and zein-based materials, mainly in the biomedical, pharmaceutical and food-packing fields. As an outline, this review consists of the introduction (Section 1), describing some of the general aspects of zein (composition, structure, characterization and film-forming properties and applications). The mixing and/or blending of zein for biomaterial applications are described in Section 2. Section 3 describes the chemical modification of zein leading to derivatives or a cross-linked 3D matrix. A description of the works dealing with the degradation and biodegradation of zein and zein-based materials is given in Section 4. The biomedical applications of zein, zein-based materials, products from the enzymatic degradation of zein and materials based on colloidal zein particles are described in Section 5. Some future trends are discussed in Section 6. Conclusions are given in Section 7. More than 130 references are cited in this review.

### 1.1. Composition and Structure of Zein

Corn contains various types of proteins, which represent about 10% of the dry mass of the grains [2]. The protein content in cereals depends on the genotype (species, variety) and the growing conditions (soil, climate, fertilization) [12,13]. Based on their solubility, corn proteins are classified into four groups: albumins (water-soluble), globulins (aqueous saline-soluble), prolamins (water-insoluble and alcohol 70%-soluble) and glutelins (water- and alcohol-insoluble) [2,14]. Corn prolamin proteins are called zeins. Most glutelins are alcohol-soluble only after reduction of the disulfide bridges and have also been classified as prolamins (zeins), due to several similarities in sequence and amino acid composition. Zein represents about 80% of the whole proteins in corn [15,16].

Zein is an amphiphilic protein, possessing both hydrophobic and hydrophilic properties. Little more than 50% of zein’s amino acid residues [17] are hydrophobic [18], including high percentages of leucine (20%), proline (10%) and alanine (10%), but zein also has a relatively high content (21%–26%) of glutamine (hydrophilic amino acid) [2].

Although being extracted from corn gluten, a renewable and relatively inexpensive source, zein is not widely produced, nor on a large scale, due to the relatively high cost of the conventional extraction process [19]. The conventional process for extracting this protein is by aqueous-alcohol solution. The extract is centrifugally clarified and then chilled to precipitate the zein. Additional extractions and precipitations increase the zein purity. After being dried, the zein forms a yellowish powder. Some studies have focused on reducing or eliminating the number and quantity of solvents in the extraction process [20,21,22]. The results of those studies have shown that zein can be extracted more effectively and at a lowered process cost as compared to the conventional process, thus making both its commercial production and use feasible [23].

Zein is classified into three different fractions, based on differences in solubility and molar mass; these fractions are called α-, β- and γ-zein [24,25]. The α-zein fraction is obtained in greater quantities in the commercial extraction process (approximately 80% of the total prolamine present in corn) and presents molar mass in the range of 21–25 kD. The β- and γ-zein fractions only made up 10%–15% and 5%–10%, respectively, depending on the genotype of the source [2].

Beyond the genotype of the source, the individual fractions and the whole protein amount in cereal grain may change with the climate and other parameters. For instance, Wroblewitz* et al.* [26] investigated the effect of CO_2_ amount on protein concentration in different grain cereals. The objective of such studies was to investigate the potential impact of the atmospheric CO_2_ concentration (expected in the near future, by the year 2050) on the quality of the grains of such cereals, mainly based on the proteic composition of several proteins. The authors verified that in maize, the α-zein fraction decreased while the γ-zein fraction rose as the maize grew at that atmospheric CO_2_ concentration. The composition, in terms of the relative proportions of zein fractions and the fine structure of zein depend also on the extraction conditions, such as the type of solvent, concentration, temperature, added reagents (such as alkali or reducing agents) and the raw material, as well as the methods of purification, concentration and drying [15,24]. The composition of the zein products affects the functional properties of zein and its uses [2]. Corn wet-milling produces a protein-rich coproduct called corn gluten meal (CGM) from which zein has been isolated commercially. Zein extracted from CGM is composed primarily of α-zein [27]. Compared to CGM, the distillerʼs dried grain (DDG) process produces low fraction of zein (29%–34%), while the CGM process produced 83% zein fraction of total proteins. In addition, α-zein isolated from CGM and DDG presented highly ordered α-helix structure [28]. In spite of the low recovery, the α-zein isolated from DDG retained its inherent characteristics, such as solubility, structure and film-forming.

**Figure 1:**
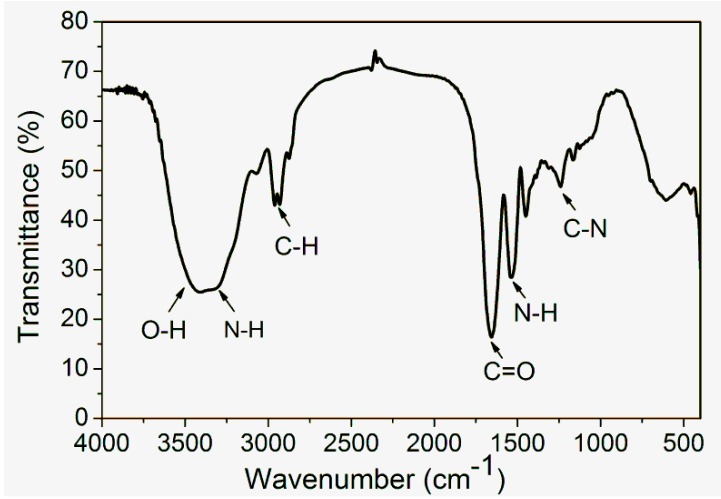
FTIR spectrum of zein, powder in KBr, 1 *w*/*w*%. Reprinted with permission from the PhD thesis authored by E. Corradini [32].

The FTIR spectrum obtained for α-zein (Figure 1) shows four characteristic bands of proteins. The band corresponding to the stretching of the N-H and O-H bonds of the amino acids of the protein appears between 2800 and 3500 cm^−1^; this band is called amide A. Another band appears at 1650 cm^−1^, corresponding to stretching of the carbonyl (C=O) of amide groups belonging to the peptide groups (amide I). The band at 1540 cm^−1^ is called amide II and corresponds to the angular deformation vibrations of the N-H bond, and lastly, the band at 1230 cm^−1^ corresponds to the axial deformation vibrations of the C-N bond [14,29].

**Figure 2:**
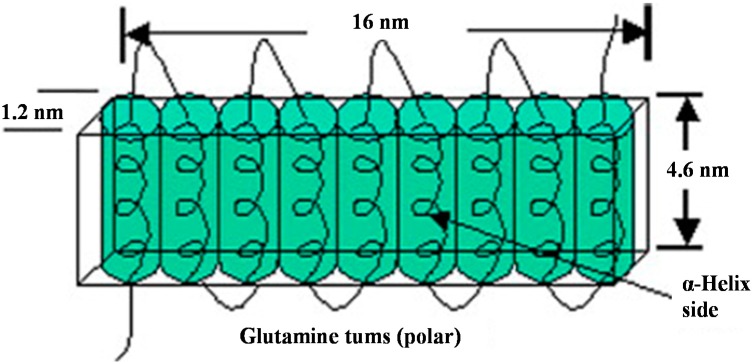
A possible structural model for α-zeins (Z22). Each of the tandem repeat units formed by a single α-helix is presented by the cylinder and glutamine-rich “turns” or loops joining them by the curve. The anti-parallel helices of tandem repeats stack linearly in the direction perpendicular to the helical axis (the *c*-axis). Reprinted with permission from [5,33]. Copyright 2008, Elsevier.

Structural models proposed for α-zein consider that this protein contains 10 successive helical segments arranged in an anti-parallel manner [30,31]. In the model proposed by Matsushima* et al.* [31], the helical segments are aligned to form a 13 nm-long asymmetric cylindrical structure (Figure 2). The sides of the cylinder correspond to the surfaces of hydrophobic helices, while the upper and lower surfaces are connected by glutamine bridges, which are hydrophilic.

### 1.2. General Properties of Zein

The solubility and chemical reactivity of zein are determined by the presence of the following functional groups: amines, amides, hydroxyls, carboxylates and phenols. The presence of these different types of groups enables zein to be physically and chemically modified to improve its functional properties. Hydrophilicity/hydrophobicity can be controlled by the addition/grafting of groups with suitable hydrophilicity. Cross-linking between zein molecules can be induced by chemicals, such as formaldehyde, glutaraldehyde, citric acid,* etc.* Due to the presence of hydrocarbon groups in zein’s side chains, zein is water-insoluble, but it is soluble in mixtures of water with aliphatic alcohols, for instance, ethanol and isopropanol, and also in other organic solvents containing hydroxyls, carbonyls, amines and other polar groups [27].

Zein is a completely amorphous polymer, which shows plasticizing viscoelasticity and a glass transition temperature (Tg) at about 165 °C, although its Tg decreases significantly in response to an increasing degree of plasticization. Lawton J. W. [34] studied the relationship between the viscoelasticity and Tg of zein and found that the addition of 20% dibutyl tartrate lowers its Tg to 60 °C. Zein is thermally stable up to 280 °C, presenting thermal degradation in a single step at temperatures beyond this value [14]. The properties of zein are dependent not only on the composition of amino acids, but also on their molecular structures at the nanometric scale. By means of atomic force microscopy (AFM), Guo* et al.* [35] found that zein in aqueous ethanol solution presents a structure composed of small globules with diameters between 150 and 550 nm. Table 1 summarizes some general properties of α-zein [12].

**Table 1:** General properties of α-zein ^a^.

Physical Form	Amorphous Powder
Glass transition temperature	165 °C
Thermal degradation point	280 °C
Molecular weight	21–25 kDa
Degree of polymerization	210–245
Isoelectric point	pH 6.2
Partial specific volume	0.771

^a^ Data source: [14].

### 1.3. Film-Forming Capability of Zein and Zein-Based Materials

Due to its molecular weight, degree of polymerization and chemical structure, zein exhibits good film-forming properties. The formation of zein film occurs through a three-dimensional network stabilized by hydrogen interactions, hydrophobic interactions and disulfide bonds between the chains of the protein.

Zein films are produced by two technological processes: a wet process based on solubilization; and a dry process based on the thermoplastic properties of zein under conditions of very low humidity. Zein films are prepared through a wet process by dissolution in a solution of aliphatic alcohols and evaporation of the solvent on inert surfaces. These films are hard, brittle, tough and usually require the addition of plasticizers for tuning some properties [36]. Several studies have been performed, targeted at understanding the effect of plasticizers on zein films [37,38,39,40]. The most effective plasticizers are those that have polar and nonpolar groups, such as triethylene glycol, oleic acid and dibutyl tartrate [41]. The viscoelastic behavior above Tg and the thermal stability allow the zein to be processed (dry process) in equipment commonly used for synthetic polymers, in the presence of a suitable plasticizer. Zein films can be obtained by processing in equipment, such as kneading, blowing and/or extrusion devices [42,43,44].

Being an alcohol-soluble protein, zein is a markedly hydrophobic material and has an excellent film-forming ability [45]. Zein’s film-forming property attracted attention in the field of edible films and coating materials [4,27]. Therefore, zein has been used to produce polymeric films that are biodegradable and can be used as a coating to protect food and related materials from spoilage. Zein films can replace commercial coating agents, like carnauba wax and shellac, inside food packets. The properties of the films, like biodegradability, mechanical properties, water absorption, barrier properties,* etc.*, largely depend on the interaction between local existent proteins, plasticizers and other functional groups. Thus, monolayers are good models for investigating and better understanding zein film properties, such as adsorption, wettability, barrier for water vapor and other gases. In this direction, Subramanian* et al.* [46] investigated the adsorption characteristics of zein on hydrophobic and hydrophilic surfaces, targeted at understand the orientation changes associated with this protein structure localized at the surface. The zein was adsorbed by a self-assembly process on a monolayer-modified gold surface. Based on the values of the initial adsorption rate and the further quartz crystal microbalance studies, the authors stated that zein shows higher affinity toward hydrophilic than hydrophobic surfaces. Based on their results, they pointed out that zein conformation changes lead to protein denaturation under ambient conditions and that the adsorption modes and conformational changes are different considering hydrophilic and hydrophobic surfaces. In this case, thermal perturbations in the dry state induced refolding of the zein structure toward a native-like state. However, under wet conditions, the zein deposits are stable and show the reversibility of conformational changes as a function of temperature. On a hydrophilic surface, the zein molecules adopt a likely perpendicular orientation, while on a hydrophobic surface, it seems to be almost flat. The studies made by Subramarian* et al.* [46] indicate the possibilities of using zein as a protective, impermeable coating for food packaging, wherein different conditions, such as hydrophilic and hydrophobic surfaces, may be required to store edible materials.

Lai and Padua [40,47] prepared films from moldable zein resins containing oleic acid as a plasticizer. The resins were prepared by stirring proper amounts of zein and oleic acid in warmed aqueous alcohol solution, then pouring the mixture into ice water to form a dough-like mass. Structural characterization of zein films by SAXS suggested a film structure consisting of staggered zein planes alternating with oleic acid layers. In a further study by the same research group [48], protein-fatty acid interactions were investigated by the use of surface plasmon resonance (SPR). In this case, studies were performed dealing with zein adsorption from water:2-propanol at a ratio of 25:75 and at conc. from 0.05% to 0.5% *w*/*v*, onto hydrophilic and hydrophobic self-assembled monolayers (SAMs) produced by 11-mercaptoundecanoic acid and 1-octanethiol, respectively. For both surfaces, the rate of initial adsorption and the ultimate surface coverage increased with the concentration of the bulk protein. The zein adsorption footprint explained the observed differences in monolayer values for hydrophobic and hydrophilic surfaces. The authors explained this observation in terms of footprint size, which, according to a current model for the molecular structure of zein, would be larger for zein binding to hydrophobic surfaces than for hydrophilic ones. Zein may have adsorbed to hydrophobic or hydrophilic SAMs utilizing different surfaces of its molecules [48].

The surface morphology and surface hydrophilicity of zein films prepared using either EtOH/water mixtures or AcOH as solvents were investigated by Shi* et al.* [49]. The surfaces were characterized using a combination of AFM, water contact angle and XPS. Furthermore, in the same paper, these authors engineered and obtained zein films of controlled hydrophilicity through the use of UV/ozone treatment. It was shown that the process efficiently decreased the water contact angles of zein films from ≈ 80° to less than 10° within 130 s of treatment. Such a result demonstrated the efficacy of the new approach to control both the surface wettability and morphology. It was pointed out that the distinct surface morphology and hydrophilicity of spin-cast zein films can be obtained by alternation of the solvents between EtOH and AcOH. In addition, they observed that zein films prepared from EtOH solutions are more hydrophilic due to the higher amount of polar functional groups at the film surface.

Beck* et al.* [3] published in 1996 a paper where the film properties of zein were deeply evaluated and compared to ethyl cellulose towards pharmaceutical applications. The authors observed that zein exhibits volumetric, water sorption and gas barrier properties, which are similar to partially-etherified celluloses in that the degree of substitution (DS) ranged from 1.1 to 1.4. Accordingly, the Tg of zein is comparatively low, which is a noticeable advantage considering the processing conditions and the final mechanical properties of zein-based materials.

Arcan* et al.* [50] published a work describing the antioxidant and antibactericidal properties on zein-based films after incorporation of different phenolic acids (gallic acid, *p*-hydroxy benzoic acid or ferulic acids) or flavonoids (catechin, flavone or quercetin). The addition of these compounds also eliminated the classical brittleness and flexibility problems associated with raw zein. Those authors emphasized that such achievements open a new perspective for using zein in flexible bioactive packaging.

### 1.4. Fibers of Zein and Zein-Based Materials

Zein also has a fiber-forming ability, which is why one of its main applications in the past was for textile fibers. The technique for producing fibers consisted of spinning alkaline solutions of zein, coagulating them with acids and salts and curing them with formaldehyde [51]. In addition, the chemicals used in such process are all environmental unfriendly; moreover, the fibers developed by this method did not present sufficiently good mechanical and water stability properties for use in textile applications. A less environmentally aggressive process for producing zein fibers to be used in textile fibers has been proposed by Uy, W.C. (1996) [52,53].

### 1.5. Fibers of Zein by Electrospinning Process

Recently, several researchers have also made efforts to obtain nanofibers for different technological applications using the electrospinning technique [6,8]. Applications of nanofibers made of zein and zein-based materials, produced by electrospinning, in biomaterial fields as scaffolds in tissue engineering or as drug carriers have been proposed, especially due to their biodegradability and biocompatibility [54]. For instance, curcumin encapsulated in zein nanofibers (with an average of 310 nm in diameter) possesses improved free radical scavenging activity and sustained release properties [55]. Li* et al.* [56] observed that the stability of (−)-epigallocatechin gallate (EGCG) was enhanced when encapsulated in zein electrospun nanofibers with an average diameter of 472 ± 46 nm after the fiber had been aged for at least one day at 0% relative humidity under ambient temperature. Mucoadhesive electrospun fibers (average diameter: 449 ± 126 nm) of a blend constituted of zein, PEO and chitosan were found to enhance mucoadhesivity [57], thereby aiding in the improved bioaccessibility and bioavailability of nutrients.

**Figure 3:**
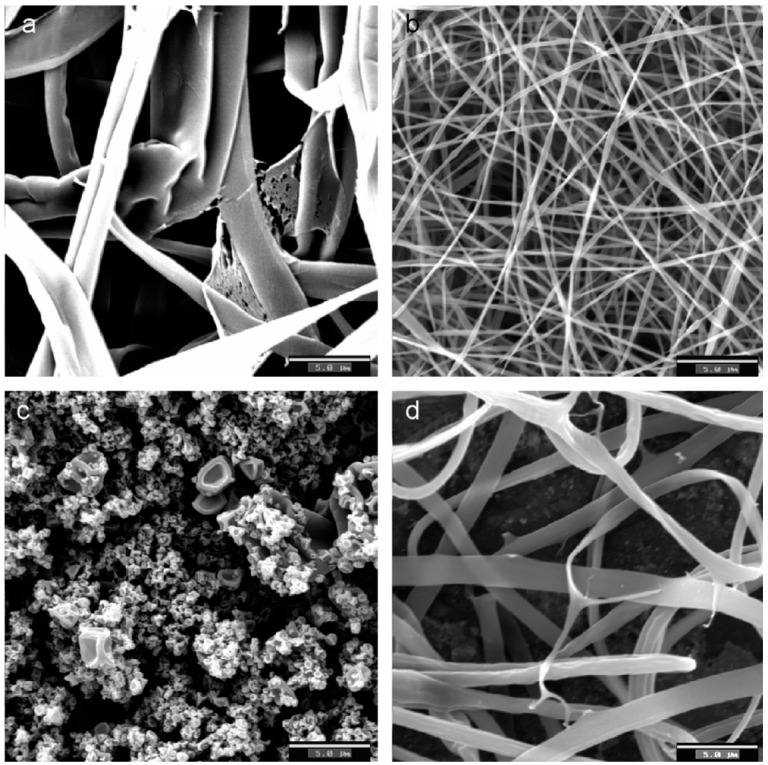
Selected SEM images of electrospun fiber networks for: (**a**) thick tubular fibers obtained from a concentrated zein solution of 50 wt %; (**b**) thin tubular fibers obtained from zein solutions with a tip to collector distance of 15 cm; (**c**) nanobeads obtained from a diluted zein solution of 12 wt %; (**d**) ribbon-like fibers obtained from the acidified zein solution. Scale markers are 5 mm in all cases. Reprinted with permission from Torres-Giner* et al.* [7]. Copyright 2007, Elsevier. Scale bar: 5.0 µm.

Torres-Giner* et al.* [7] studied the effect of process parameters, such as injection speed, concentration of the solution, voltage and needle tip to collector distance, in the production of zein fibers by electrospinning. The results showed variations of 100 nm up to 1 μm in diameter and significant differences in the morphology of the fibers as a function of processing conditions. The zein fibers showed a tendency to form a tubular morphology (Figure 3), although other more complex morphologies, such as nanobeads and ribbons, were also observed. ATR-FTIR spectroscopy revealed that the secondary structure of zein, particularly the length of the α-helix, differs depending on the electrospinning conditions employed, and this is expected to influence the final properties of the fibers.

Nanofibers of zein were produced by Yao* et al.* [58] through electrospinning combined with cross-linking to improve the mechanical properties of the spun mats. Zein was electrospun from aqueous ethanol solutions, leading to the formation of nanoparticles, nanofiber mats or ribbon-like nanofibers. The as-obtained zein nanofiber mats were further cross-linked by contact with hexamethylene diisocyanate (HDI). The tensile strength of electrospun zein nanofiber mats increased from 1.7 MPa (uncross-linked) to 4.2 MPa (cross-linked) if the concentration of zein is 40% *w*/*v* in an ethanol/water solution 70/30 *v*/*v* [6]. Nanofibers of zein blends through electrospinning have been performed by several authors. This is a good strategy for changing the properties by adequately choosing the blend constituents and also the polymer ratio. This subject will be discussed in Section 2.1.

## 2. Mixing or Blends Based on Zein Aimed at Biomaterial Applications

### 2.1. Blends of Zein with Polymers

Due to its properties, such as thermoplasticity, good gas barrier, biocompatibility and biodegradability, zein has proven to have potential for uses combined with other polymers to produce new materials for applications in various areas, such as biomedicine, pharmaceutical products and packaging. Its amphiphilic polymeric nature makes zein highly versatile to be joined with both hydrophilic and hydrophobic polymers in the production of compatible materials with better properties than the individual components. The literature reports on several studies about blends of zein with conventional synthetic polymers, such as polyethylene, nylon and poly(vinyl pyrrolidone) (PVP), and with biodegradable (natural or synthetic) polymers, such as starch, poly(ε-caprolactone) (PCL),* etc.*, retaining the biodegradability character of zein.

Several studies involving zein and starch blends are reported in the literature [2,59,60]. These studies indicated that equilibrium water absorption decreases with increasing zein content in the blend. The addition of zein favors the rigidity of the blends, increasing their modulus of elasticity and tensile strength and reducing their deformation. The blends were immiscible, showing two distinct starch-rich and zein-rich phases. The presence of cracks and flaws in the interfacial region indicated little interfacial adhesion or only one type of physical interaction between the phases. Habeych* et al.* [61] modified starch by oxidation, using sodium hypochlorite in the presence of the 2,2,6,6-tetramethylpiperidine-1-oxyl (TEMPO) radical and NaBr to prepare aldehyde starch. The results obtained through microscopy and tensile tests indicated that the blends had improved adhesion between the zein and modified-starch phases, probably due to the reaction between the aldehyde groups in the starch molecules and zein. Leroy* et al.* [62] evaluated the potential of an ionic liquid (1-butyl-3-methyl imidazolium chloride, [Bmim]Cl, used as a multifunctional plasticizer) for starch and its blends with zein. Their results indicated that, compared to glycerol, the use of [Bmim]Cl leads to a less hygroscopic material, a more efficient plasticization of both the starch and zein phases and the compatibilization of starch/zein blends.

Blends of poly(ε-caprolactone) (PCL) with zein (PCL/zein) have also been prepared by melt-processing PCL/zein blends, showing reduced tensile strength and elongation at break, but higher Young’s modulus than pure PCL. These mechanical properties were indicative of an incompatible system [63]. Sessa* et al.* [64] prepared blends of zein and poly(vinyl pyrrolidone) (PVP) by melt processing. They found that the presence of PVP in the blend with zein led to a significant increase in tensile strength, indicating that the Zein/PVP blends are compatible. Blends of zein with nylon-6 have been produced by solution casting of the film, using formic acid as the solvent [65]. When the amount of nylon-6 was 8% or less, the as-formed blends exhibited greater tensile strength and lower solubility.

Li* et al.* [66] reported the use of Pluronic F127 triblock copolymer to improve the flexibility of zein film. The composite films were prepared by casting, and the content of Pluronic was varied in the composites. The results indicated that different F127 loads cause strong impact on the physical properties of composite films, which arise from the competing effects of crystallization and plasticization of F127. At relatively low F127 loads (0% to 35%), the plasticizing effect predominated, and the elongation at break of zein composite film containing 35% F127 was about eight-fold higher than that of the zein film with 10% F127. At high F127 loads (50% or higher), a large number of lamellar crystals were formed in the film matrix, which overcame the plasticizing effect [66].

Few studies reported in the literature have focused on the development of zein microparticles combined with other biodegradable polymers for controlled drug release. Chen (2009) produced microspheres of soy protein isolate (SPI), zein and SPI/zein blends using the cold gelation technique [63]. The kinetics of the release of microspheres was investigated in simulated gastrointestinal fluid, using riboflavin as a nutrient model. Zein microspheres showed lower release than soy microspheres. The release rate decreased progressively, increasing the zein content in the SPI/Zein microspheres. Blending of SPI and zein proved to be a suitable method to control the release of hydrophilic nutrients, such as riboflavin, from microspheres [67].

Microspheres of poly(d,l-lactide-*co*-glycolide) (PLA) and zein were prepared by spray drying to release amoxicillin (AMX) for root canal disinfection. The release of AMX from microspheres depends on the PLGA/zein composition. According to the authors, the drug levels achieved in the intracanal dressing were effective in between visits during endodontic treatment [68]. Müller *et al.* [69] synthesized microspheres based on zein and zein associated with chitosan. Their study showed that the addition of chitosan increased the porosity of the microspheres when compared to free-chitosan zein microspheres. Their chemical and physical characterization and morphological analysis led them to infer that zein/chitosan microspheres are good candidates to act as carriers for controlled drug release.

The studies described show that the association of zein with other polymers is of great importance for the development of new biodegradable materials. However, few studies have measured the (bio)degradability of such materials using specific methods, such as the amount of CO_2_ produced or the changes in the molecular mass as a function of time. Some results relative to the degradability of zein and zein-based materials are described in Section 4 of this review.

### 2.2. Zein-Blended Nanofibers Performed through Electrospinning

The studies cited in last section involved the production of zein-based multiphase materials by casting and melt processing methods. However, fibers of zein-based blends have been also prepared by the electrospinning method, as already mentioned in Section 1.4, to obtain fibers with diameters ranging from the nano to micrometric scale for biomedical applications, mainly as scaffolds in tissue engineering and as vehicles for drug release. In some studies, zein has been added to other polymers to facilitate the electrospinning process. One key challenge in this case is to find a solvent or solvent mixture in which the polymers are soluble. Zein/silk fibroin (SF) blends were electrospun to produce nanofibers, using formic acid as the solvent. It was observed that the fiber diameter decreases from 265 to 230 nm with increasing SF content in the blend. The tensile strength of the fibrous zein/SF membranes was improved by increasing the SF content in the blend. Preliminary experiments of* in vitro* degradation and cytotoxicity of zein/SF fibrous membranes indicated that these fibrous membranes have potential applications as biomedical materials [70].

Zein/chitosan fibers were prepared by electrospinning, in proportions of 99/1, 97/3, 95/5 and 90/10 (*wt*/*wt*), in a solvent mixture of ethanol/trifluoroacetic acid (TFA) 2:1 (*wt*:*wt*). Variations were observed in both fiber morphology and diameter as a function of the proportion of chitosan in the blend. The fibers thus obtained were water-insoluble and exhibited antimicrobial activity, which was attributed to the presence of chitosan (even in low amounts, up 10 *wt* %). These fibers show a proven potential for use in pharmaceutical and biomedical applications [71].

Nanofibrous membranes for wound healing were developed by collagen and zein electrospinning in aqueous acetic acid solution. The combination of zein was found to improve the electrospinnability of collagen. It was found that the properties of electrospun membranes (fiber diameter, surface wettability, mechanical and* in vitro* degradable properties, as well as cell adhesion ability) could be modulated by the collagen/zein composite. Incorporating the drug berberine into the blend before the electrospinning process showed the efficient antibacterial wound healing properties of the as-obtained nanofibers. When used as a dressing covering full-thickness skin wounds in mice, the as-prepared nanofibrous membrane was observed to induce fast tissue regeneration [72].

Antibacterial electrospun scaffolds were prepared by physically mixing polyurethane (PU) with cellulose acetate (CA) and zein for wound dressing applications. PU was used as the main polymeric component; CA and zein were added to achieve desirable properties, such as better hydrophilicity, excellent cell attachment, proliferation and blood clotting ability. Streptomycin sulfate, an antimicrobial agent, was incorporated into the electrospun fibers to prevent common clinical infections, and its antimicrobial activities against Gram-negative and Gram-positive bacteria were examined. The* in vitro* antimicrobial activity of the nanofiber membranes was evaluated for use in wound dressings. The results indicated that cells interacted favorably with the composite scaffold. Moreover, the composite material showed good bactericidal activity against both Gram-positive and Gram-negative bacteria. The PU-CA-zein-drug composite scaffolds showed better blood clotting ability than pristine PU nanofibers. The presence of CA and zein in the nanofiber membrane improved its hydrophilicity and permeability to air and moisture. CA and zein not only increased the uptake of liquid, but also created a moist environment for the wound, which can accelerate wound recovery. The authors claimed that this composite material could be an ideal biomaterial for wound dressing [73].

### 2.3. Mixing Zein or Zein-Blends with Non-Polymer Moieties

Several studies have been published regarding the development of zein composites or zein blends with inorganic materials for bone regeneration purposes. For instance, Zhi-Hu Qu* et al.* (2008) combined hydroxyapatite with a zein matrix to produce composite porous scaffolds via salt leaching technique for bone tissue engineering (BTE) applications. Based on* in vitro* tests with human bone marrow stromal cells, the scaffolds proved be able to support mesenchymal stem cell (MSC) adhesion, proliferation and osteogenic differentiation. Based on both mechanical and biological assessments, the HA-coated zein scaffold was found to be optimal biomaterial for bone tissue engineering [74].

Salermo* et al.* [75] developed a multiphase material for the fabrication of 3D porous scaffolds to be used in bone regeneration. The material composed of PCL, thermoplastic zein (TZ) (a thermoplastic material obtained by mixing zein with poly(ethylene glycol)) and microparticles of osteoconductive hydroxyapatite (HA) was prepared by melt mixing. Compared to neat PCL, PCL-HA composite and PCL/TZ blends, the multiphase PCL/TZ/HA showed improved mesenchymal stem cell (MSC) adhesion, proliferation and osteogenic differentiation ability [75]. In another study, Salermo* et al.* [76] designed a multiphase porous scaffold based on PCL, TZ and HA via supercritical CO_2_ foaming. The porosity of the materials was controlled by the foaming temperature and formulation parameters. The results obtained from tensile tests,* in vitro* degradation and biocompatibility assays for PCL-TZ-HA composite scaffolds revealed adequate properties for bone tissue engineering (BTE) [76]. Novel multiphase biomaterials for BTE were designed and fabricated by Salermo (2012) by blending PCL, TZ and HA. The materials were then characterized to assess their morphological and microstructural properties, wettability,* in vitro* degradation and biological response. The TZ improved the wettability of PCL and accelerated the degradation of the PCL/TZ blend, and the three-phase PCL/TZ–HA composite became almost completely degraded after 56 days of incubation in PBS. However, the tensile properties of the biomaterials were significantly lower than those of PCL. The simultaneous addition of TZ and HA particles into PCL induced a significant increase in the osteogenic properties of the materials, by causing the MG63 cells to deposit calcium by one order of magnitude higher than PCL [77]. Porous scaffolds of zein/PCL biocomposite were prepared by the solvent casting and particulate leaching method, using sodium chloride particles as the porogen. The addition of zein to PCL increased the hydrophilicity of the composite, with an improvement in cell adhesion and proliferation. *In vitro* degradation tests showed that zein/PCL scaffolds degraded faster than PCL scaffold and that the degradation rate could be controlled by the amount of zein in the composite [78].

## 3. Some Methods for Chemical Modifying Zein

New technologies are needed for modifying and processing materials from renewable resources to develop new biodegradable materials, providing similar or better properties compared to existing ones. Among renewable resources, proteins have long been used. Most proteins contain hundreds of amino acids linked in a specific sequence, which gives a certain protein its inherent properties. The secondary, tertiary and quaternary structures of proteins result in various interactions and bindings differing in position, type and energy [1]. As zein constitutes *ca*. 80% of the whole protein matrix in corn, it is considered a good example of a proteic material from a renewable resource. The average molar weight of zein is 44 kDa, so zein possesses a wide variety of reactive side groups, such as amide (53%), amine (1%), carboxyl (4%), hydroxyl (24%) and phenolic (8%), due to the amino acids as constituents of the main chain [79]. Anderson and Lamsal [15] published in 2011 a review describing several works in which cross-linking of zein was performed in different conditions. The combination of chemical modification and physical processes can be used for changing the zein properties as compared to the raw material. Different chemicals, such as formaldehyde, glutaraldehyde and epichlorohydrin, can be used for reacting with zein, resulting in a cross-linked material. In this direction, Selling* et al.* [80] prepared, for the first time, cross-linked zein using glyoxal (GLY) as the cross-linking agent via reactive extrusion using a twin screw extruder, a well as dilute sodium hydroxide as catalyst and tri(ethylene glycol) (TEG) as plasticizer. Compression or injection molding was used to process the ground extrudate. In a given formulation (GLY = 6% and TEG = 10%), samples could be obtained from the injection mold; however, they did not hold their molded shape due to the sample’s elasticity at the mold temperature. In another formulation using lower levels of GLY, the authors obtained injection molded sample bars possessing similar quality to the control (0% of GLY). Parris and Coffin [37] prepared cross-linked zein in different solvent conditions. They saw that when the reaction is performed in aqueous ethanol, the tensile strength and modulus significantly increased over the control (unmodified zein). However, in aqueous acetone, the as-produced films presented poor mechanical properties as compared to the control.

Biswas* et al.* [81] developed a novel method to derivatize the surface of zein, allowing a change in the water absorption and surface wetting capabilities. They used octenyl succinic anhydride and alkyl and alkenyl ketene dimers. The authors pointed out that the proposed methodology is easy to apply and includes a step with baking with derivatizing agent at an appropriate concentration. The as-obtained materials presented lower water absorption and, due to the hydrophobic character, led to an increase of the water contact angle relative to the control, demonstrating the advantages of this methodology. The derivatized surface of the film became very different, as observed by atomic force microscopy, possessing larger globular domains extending up to 122 nm above the lowest surface.

Braeuer* et al.* [82] used acylation reactions to chemically modify native plant proteins, such as gluten, zein, soy and pea protein, by reaction with palmitic acid chloride and alkenyl-substituted succinic anhydrides. The authors targeted developing novel, chemically-modified protein materials, which remain biodegradable and processable by thermoplastic shaping in extruders. The biodegradability of the acylated protein derivatives was demonstrated by the authors. They concluded that the chosen plant proteins are suitable for acylation reacting with accessible amine and hydroxyl groups of the plant proteins to form new amide and ester bonds. This leads to fusible thermoplastic materials with improved resistance to water. However, the resultant extruded articles are brittle, combined with a low tensile strength. The addition of glycerol up to 10% conc. improved the processability and mechanical performance of the acylated products. Senna* et al.* [83] studied the effect of gamma irradiation and graft copolymerization with different ratios of acrylic acid monomer (AAc) on the improvement of zein and poly(vinyl alcohol) (PVA) compatibility. Tensile tests showed that the addition of PVAl increased the flexibility of the blends. SEM micrographs indicated that gamma irradiation and graft copolymerization reduced the interfacial tension between the phases in blends and improved the dispersion of the polymers, thus enhancing the compatibility of zein/PVA blend films.

Although the addition of plasticizers, such as glycerol, polyol and fatty acid, diminishes the brittleness of zein films, these plasticizers may facilitate the absorption of moisture from highly humid atmospheres, impairing the barrier and mechanical properties of zein films. Other alternatives have been sought to improve the water resistance and toughness of zein-based films for packaging applications. Cross-linking between zein and chemical reagents, such as formaldehyde, glutaraldehyde, epichlorohydrin, citric acid, butane-1,2,3,4-tetracarboxylic acid, polymeric dialdehyde starch, 1,2-epoxy-3-chloropropane and glyoxal, have also been reported. The cross-linking of zein has resulted in significantly increased mechanical strength, but often has negative effects on flexibility. Wu (2003) studied the chemical modification of zein with PCL and hexamethylene diisocyanate (HDI) prepolymer. The modified zein was plasticized using dibutyl l-tartrate (DBT) and then compression molded. The incorporation of PCL and plasticization with DBT in compression molded zein sheets improved water resistance, tensile strength and elongation [84]. Shi* et al.* [85] investigated the modification of zein with lauryl chloride through an acylation reaction. The mechanical and water absorption properties of the chemically-modified materials were investigated as a function of lauryl chloride content. The elongation at break of the modified zein sheet was enlarged by about seven-fold at the high modification level, even though with some loss of mechanical strength. The surfaces of modified zein films were more uniform than the unmodified zein film, but became more hydrophobic. Based on this fact, the authors suggested that no microphase separation occurred during the film formation process.

Unfortunately, only a few works dealing with the chemical modification of zein have been found in the literature. This means that an opportunity remains opened for researchers targeting the development new materials based on the reactive properties of the functional groups of zein’s amino acids.

## 4. Biodegradation of Zein and Degradation of Zein and Zein-Based Materials

### 4.1. Biodegradation of Zein

As mentioned, the (bio)degradation of zein and its coproducts is a very important aspect that determines the use of such materials in specific applications. Some studies have been performed for evaluating and understanding the biodegradation of zein. For instance, Iman and Gordon [86] evaluated the biodegradation of zein and coproducts from industrially processed corn, corn fiber, corn zein, cornstarch, distillers’ grain and corn gluten meal in compost environments under changeable temperature, pH and moisture conditions. The authors observed that, generally, composts with higher temperature (40 °C), neutral pH (7.0) and 50%–60% moisture seemed to be ideal for corn coproduct biodegradation, particularly for corn gluten meal and corn zein. Low moisture conditions, however, slowed considerably biodegradation, but as the moisture content was increased up to 60%, the degradation rates were improved. Thereafter, increasing the moisture factor particularly slowed the degradation of corn gluten meal and corn zein, whereas cornstarch degradation remained unaffected [86]. Very important challenges that remain for biodegradability in soil is the difficulty in transferring biodegradability results to different soils and climates and also the lack of validation tests through a positive reference and, also, setting the prerequisites for soil media. These aspects were described in the review published in 2010 by Briassoulis and Dejean [87] in which a critical review of the existent norms and standards for the biodegradation of plastics used in agriculture is made.

### 4.2. Degradation of Zein and Zein-Based Materials

Being a protein, zein can be degraded in enzymatic media. Among the enzymes (proteases) that may be used as catalyst for zein degradation, the following can be cited: pepsin [88], thermolysin [89] and trypsin [90]. A study performed by Hurtado-Lopez and Murdan [88] showed that zein microspheres were extremely resistant to degradation in the absence of enzymes, but in simulated gastric and intestinal fluids, zein microspheres were degraded by pepsin and pancreatin enzymes, respectively. Mannheim and Cheryan [91] used different enzymes to obtain water-soluble modified zein. They used alcalase and SP-369 (an experimental, thermally-stable bacterial protease), pronase (a mixture of endo- and exo-peptidases prepared from *Streptomyces griseus*), papain and Milezyme APL 440 (a serine protease from *Bacillus licheniformis*)*.* In such a protocol, organic solvent and protease were used for partially hydrolyzing zein in a first step, followed by hydrolysis in aqueous solution having the same protease. A significant increase in solubility from 0% (for the unmodified zein) to 99% (for enzyme-modified and ultrafiltered zein) was observed. Of course, the process used decreased the molecular weight distribution compared to unmodified zein.

Fujimaki* et al.* [92] investigated the degradation of zein during the germination of corn and gave special relevance to protease activity. Using polyacrylamide gel electrophoresis, the authors observed that, as the germination carried on, zein was rapidly degraded into two main subunits. The protease’s activity increased remarkably during the germination. For the third day of germination, the main amino acids found were leucine, alanine and glutamine. At the same time, the authors observed that free amino acids, especially phenylalanine and tyrosine, were formed during zein degradation. Based on these results, the authors suggested that there is a proteolytic system in which the aromatic amino acids can be liberated more effectively than others. The authors prepared a crude extract of protease that degrades zein* in vitro* to form relatively large amounts of free phenyl alanine and tyrosine. The protease’s activity enhanced when in the presence of 2-mercapto ethanol. The authors concluded that the degradation of zein during germination is probably conducted by sulfhydryl protease. Bobokalonov* et al.* [93] used an enzyme to degrade microgels constituted of low methylated pectin/zein (at various P/Z ratios) loaded with piroxicam. In this case, they used HCl/KCl, 0.2 M, pH 1.2, buffer solution containing the hydrolytic enzyme pepsin for modeling the stomach milieu; and phosphate buffer, 0.2 M, pH 6.4, for modeling the intestinal milieu.

## 5. Biomedical Applications of Zein-Based Materials

### 5.1. As Drug Carriers for Drug Delivery

Zein-based micro- and nano-composites present good applications in the drug release field, due to their excellent biocompatibility, biodegradability and high surface contact [94,95]. Zein can interact efficiently with hydrophobic and hydrophilic drugs, acting as delivery vehicles [17]. Because of its high percentages of hydrophobic amino acid residues, zein is insoluble under physiological conditions and capable of the sustained release of encapsulated compounds [88]. Additionally, the high glutamine content in the terminal structure increases the polarity of zein chains [96]. Therefore, the hydrophobic regions of the protein promote the aggregation of colloidal particles, and the polar side chains can interact with DNA [97]. Zein degradation occurs through a lot of enzymes and has been shown to be especially well suited for oral delivery [98,99]. Furthermore, part of the *N*-terminal region of zein interacts with cell membranes, acting as a peptide carrier for drugs across cell membranes [100]. However, drug carrier systems of zein-based materials are applied mainly as vehicles for the delivery of hydrophobic drugs, since these components present poor absorption and low bioavailability and, therefore, cannot be absorbed by the body [97]. Zein acts as an intermediary agent between the hydrophobic drug and epithelial cells of colon, since this protein presents a good hydrophobic-hydrophilic property, a fact that increases the mucoadhesion of zein-based materials with the epithelial cells in the gastrointestinal tract. Therefore, zein increases hydrophobic drug permeation and absorption, acting as a targeted delivery and controlled release agent [97].

Li* et al.* [101] investigated a preparation of zein/ibuprofen (IBU) microcomposites (zein/IBU) using a modified electro-spraying process. The DSC curve of hydrophobic IBU drug exhibited a single endothermic peak at 77 °C, attributed to the melting point. However, zein/IBU microparticles did not show any fusion peaks or phase transitions in the DSC curves, suggesting that IBU was no longer present as a crystalline material and was converted into an amorphous state in the microcomposites [101]. In this case, the H-bond interactions among zein-IBU allowed the formation of amorphous composites, a fact that favored IBU release from zein/IBU particles. Therefore, the release assays of IBU from zein/IBU systems demonstrated that the microparticles provided better sustained drug release profiles, and this modification can be beneficial in the development of advanced pharmaceutical materials [101]. Hu* et al.* prepared zein/lutein (zein/LUT) nanoparticles from solution-enhanced dispersion by supercritical fluids (SEDS) [102]. Lutein (LUT), a natural pigment widely found in fruits, vegetables, flowers and some algae, has been applied in the prevention of cardiovascular disease, stroke, lung cancer and breast cancer [19,103]. However, LUT is unstable against light and heat. However, it presents high crystallinity, low-water solubility, poor absorption and low bioavailability [102]. Therefore, Hu* et al.* [102] protected the LUT from light degradation by encapsulating the drug into zein nanoparticles, improved its aqueous solubility and enabled specific drug delivery in the colon region. According to Hu* et al.*, the interactions among zein-LUT provided the existence of an amorphous state in the nanoparticles [102]. Therefore, the interactions among zein-hydrophobic drug significantly altered the drug physicochemical properties. The LUT release behaviors from zein/LUT nanoparticles were investigated and compared to raw LUT and a physical mixture of LUT and zein, as control samples [102].

The LUT dissolution is faster from both raw LUT and a physical mixture of zein-LUT than from zein/LUT nanoparticles. Within the first 5 min, more than 70% of the drug was released from both raw LUT and the physical mixture samples, while only about 15% from the nanoparticles [102]. Further investigation of the LUT release in the first 40 min was carried out. The release profile of the drug from raw LUT is very similar to that of the physical mixture. However, the release rates from both of them are also faster than that of the nanoparticles. Moreover, the initial burst release of LUT was hardly observable for the nanoparticles, with the release profile displaying a near zero-order release, a desired property for controlled release devices [102]. The mechanism of LUT release from nanoparticles might be controlled by swelling/erosion of zein nanoparticle matrices. After 40 min, the release rate got slower, most likely due to the long diffusion route of LUT entrapped deeper in the zein nanoparticles [104].

Luo* et al.* [105] obtained nanoparticles of zein and zein/carboxymethyl chitosan (zein/CMCS) through liquid-liquid phase separation, associated with the ionic gelation technique. Indole-3-carbinol (I3C) and 3,3'-diindolylmethane (DIM) are hydrophobic, bioactive compounds extracted from cruciferous vegetables that have good potential for cancer treatment. However, I3C and DIM present low stability in gastric conditions and degrade when exposed to light and heat [105]. Under acidic conditions, I3C and DIM molecules undergo oligomerization, forming a mixture of compounds known as acid condensation products. Therefore, the nanoparticles of zein and zein/CMCS were utilized with the aim of protecting labile compounds from harsh conditions, as well as providing controlled release and targeted delivery of drug bioactives [105]. The studies showed that both nanoparticle formulations provided controlled release of these bioactive compounds. The I3C and DIM stabilities were significantly improved after encapsulating in nanoparticles. Both zein and zein/CMCS nanoparticles provided good protection against degradation in UV-light. On the other hand, under the studied thermal conditions, zein/CMCS nanoparticles provided better protection of I3C against degradation and oligomerization [105].

**Figure 4:**
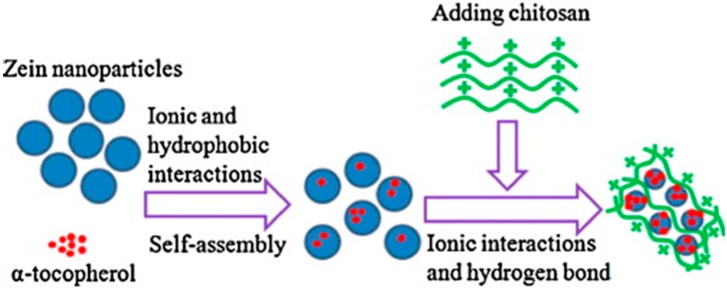
Schematic illustration of the formation of the zein/chitosan complex for encapsulation of α-tocopherol (TOC). Reprinted with permission from [106]. Copyright 2011, Elsevier.

Luo* et al.* [106] also obtained zein nanoparticles complexed with chitosan containing α-tocopherol (zein/CS/TOC), as shown in Figure 4. In this case, zein nanoparticles improved the stability of α-tocopherol (TOC), and the chitosan (CS) coating did not affect the encapsulation efficiency, but greatly improved the controlled release properties of TOC in PBS buffer solution, indicating that nanoparticles of zein coated with CS can be developed as a novel TOC supplementation or treatment [106]. The association of zein with CS was investigated by Muller* et al.* In this case, zein and zein/CS microparticles were obtained and applied as a carrier matrix for controlled drug release [69]. Bobokalonov* et al.* [93] applied zein/pectin microspheres as drug delivery systems and studied the release kinetics of piroxicam drug from zein/pectin microparticles. The kinetic parameters of piroxicam release were calculated from Peppas’s model [107] and by a first-order equation model [93]. The results showed that the diffusion of piroxicam (a therapeutic agent) was the rate-limiting step in drug release from the microspheres. For this same purpose, Mehta* et al.* [108] encapsulated anti-tuberculosis drugs in zein-based microspheres. Zein-microparticles increased the stability of rifampicin, isoniazid and pyrazinamide drugs, enabling the controlled release of such drugs following the first-order kinetics model. Zein microparticles were also utilized as carrier of antimicrobials drugs to improve the long-term effectiveness of antimicrobials in foods and to prevent the direct contact of the drug with the food. Zein microcapsules were obtained from spray-drying at different temperatures, and the* in vitro* release kinetics of nisin was evaluated [109,110]. Nisin is a well-known bacteriocin with 34 amino acids and a molecular weight of 3510 Da. Numerous studies reported that the antimicrobial activity of nisin is reduced when it is applied in foods, possibly due to binding with food matrix components, which makes the antimicrobial unavailable to inactivate microorganisms. Nisin interacts with zein, mainly through H-bond forces, and whether the release of such drug is favored in a certain condition, the release profiles can be used as guidelines to identify the applicability of a particular nisin delivery system in a given food product [109,110].

**Figure 5:**
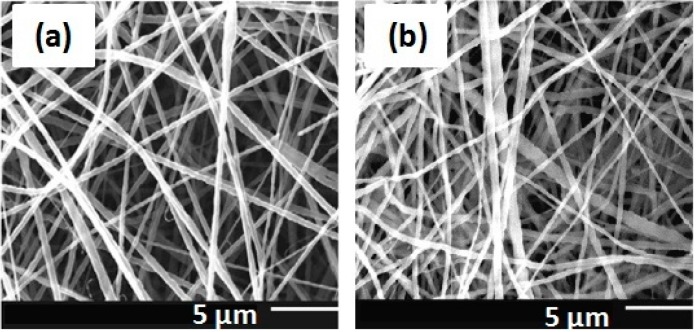
SEM images of zein/PEO/CS composite electrospun fibers with (**a**) and without α-tocopherol (**b**). Reprinted with permission of [57]. Copyright 2014, Elsevier.

Wongsasulak* et al.* [111] prepared nanofibers based on zein, poly(ethylene oxide) and CS (zein/PEO/CS thin fibers; the micrograph is presented in Figure 5a) with a good mucoadhesion property in the gastrointestinal tract. This property is very important in the development of new drug carrier systems with targeted drug release. The gastro-mucoadhesion was initiated by wetting and swelling of the polymeric molecular chains of fiber, which led to a molecular interaction between the fiber and mucin molecules (protein of epithelial tissues). Posteriorly, Wongsasulak* et al.* [57] investigated the effects that an encapsulated hydrophobic drug, such as TOC, would have on the gastro-mucoadhesion property of electrospun fibers of zein/PEO/CS (images in Figure 5). It was observed that the TOC did not affect the fiber morphology (Figure 5b), but significantly enhanced the zein/PEO/CS mucoadhesive property [22]. On assays performed at pH 1.2, in the presence of pepsin the TOC release was triggered by erosion, probably through degradation of zein chains; and at pH 2, without pepsin, the TOC release was triggered by swelling and driven by diffusion [57].

Wang and Chen [112] developed nanofibers based on hordein/zein proteins. The fibers containing 30 *wt* % of zein exhibited a stable assembled network structure, good stability in water and good tensile strength. The release assays indicated that the fibers with a 3D porous structure could serve as carriers for the controlled release of incorporated bioactive compounds (as, for example, riboflavin) in PBS buffer solution. The authors observed that the fibers were pepsin resistant and stable in simulated gastric fluid (SGF), whereas they could be digested in a simulated intestinal fluid (SIF) to gradually release the incorporated compounds where they are normally absorbed [112]. In a further work [113], the same research group obtained the same material applying two physical approaches: (i) incorporation of surface-modified cellulose nanowhiskers (SCNs); and (ii) fiber alignment, to reinforce the assembled hordein/zein electrospun nanofabrics. The stability and mechanical properties of the modified fibers were evaluated in relation to the fiber morphology, and the structure was characterized by SEM, TEM, FTIR and Raman spectroscopy. It was concluded that the SCNs modified by quaternary ammonium salt were well-dispersed in hordein/zein networks, significantly improving the mechanical properties and water resistance.

### 5.2. As Scaffolds in Tissue Engineering

Reconstruction or regeneration of hard tissue (such as bone and cartilage) using tissue engineering techniques requires the use of temporary porous scaffolds within which the cells are seeded and cultured* in vitro* before implantation [114]. The scaffolds should provide an appropriate environment for cell differentiation, proliferation and the formation of new tissue [115]. Scaffolds for bone tissue engineering should satisfy some requirements. Appropriate porosity, pore size and pore structure are necessary to facilitate the ingrowths of cells and the formation of new tissue. The scaffolds should possess good biodegradability and a suitable degradation rate to match the new tissue formation rate [114,115]. Good biocompatibility and a suitable mechanical property are also required [114]. Currently, natural-based biopolymers, mainly proteins (zein, collagen, gelatin and silk) and some polysaccharides (starch, alginate, cellulose, chitosan,* etc.*), are primary candidates for the design of bioactive composites for bone tissue engineering applications, owing to their similarity with some of the most important organic components of the extracellular matrix of native bone [114]. On the other hand, associating these biopolymers with others components, such as poly(ε-caprolactone) (PCL) [76,116], hydroxyapatite particles (HA) [117,118] and polyurethane (PU) [73], may improve the mechanical and biological response of scaffold materials.

Wu* et al.* [78] studied the fabrication of zein/poly(ε-caprolactone) (zein/PCL) porous scaffolds. Porous biocomposite scaffolds with a porosity of around 70% and a well-interconnected network were obtained. Zein led to the improvement of hydrophilicity as indicated by the results of drop-water contact angle measurement, and the degradation rate of the scaffold could be tuned by adjusting the zein content in the composite. The results demonstrate the potential of the zein/PCL biocomposite scaffolds to be used in tissue engineering strategies to regenerate bone defects [78]. Biodegradable porous scaffolds of PCL/thermoplastic zein (ZT) (or PCL/ZT scaffolds) were obtained from a supercritical CO_2_ (scCO_2_) foaming process, being the multi-phase blend constituted by water/oil, with the oil phase composed of PEG400 (special for bone tissue engineering). In this case, the HA particles (conc. of 10 and 20 *wt* %) were inter-dispersed into PCL/ZT scaffolds [76]. Modulating the material formulation and foaming temperature (T_F_) controlled the scaffolds’ porosity. Morphological, micro-structural and biodegradation analyses of the scaffolds were performed, as well as* in vitro* biocompatibility tests. Results demonstrated that both HA concentration (10 and 20 *wt* %) and T_F_ significantly affected the microstructural features of the scaffolds. In particular, scaffolds with suitable porosity and a pore size distribution, mechanical properties and biodegradability adequate for bone tissue engineering were designed and produced by selecting T_F_ equal to 100 °C for all of the used compositions. The* in vitro* cell/scaffold interaction study also demonstrated that the proposed scaffolds allowed for the adhesion and colonization of pre-osteoblast MG63 and hMSCs cells.

In further work, Salerno* et al.* [116] proposed preparing bi-modal porous scaffolds for bone tissue engineering (BTE) by combining supercritical CO_2_ (scCO) foaming and porogen leaching techniques. The factors, such as saturation, temperature, pressure and depressurization time, were selected to induce pore formation and to optimize the pore structure of the foams. As a consequence, a macro-porosity suitable for bone cell colonization and adhesion was obtained. It was demonstrated that the proposed technique contributed to the formation of PCL/TZ and PCL/TZ-HA composite scaffolds via a green chemistry process. In this case, the scaffolds showed macro-porosity between 20 to 400 µm. *In vitro* assays showed that the scaffolds induced the colonization, cell adhesion and proliferation during 28 days, demonstrating potential for bone tissue engineering.

In two other studies, Salerno* et al.* [77,119] prepared scaffolds of PCL/ZT, ZT/HA, PCL/HA and PCL/ZT/HA composites. The authors observed that zein also improved the wettability of PCL and accelerated the degradation of PCL/ZT and PCL/ZT/HA composites. However, the tensile properties of the biomaterials decreased significantly compared to PCL scaffolds. The simultaneous addition of ZT and HA particles into PCL induced a significant increase of the osteogenic properties of the materials, by causing a magnitude higher calcium deposit by the osteoblast cells, relative to pure PCL. The good selection of the experimental conditions allowed the design of multiphase 3D porous scaffolds suitable for bone tissue engineering.

In two different works, Yao* et al.* [117] and Zhang* et al.* [118] obtained nanofibers of zein/HA with a reasonable tensile strength and applied these materials as scaffolds for tissue repair. Osteoblast adhesion tests and* in vitro* cytotoxicity assays showed that the mineralized zein nanofibrous membranes had a positive effect on osteoblast growth and did not induce cytotoxic action. The zein/HA membrane presents unique nanofibrous structural features, and a coating of HA nanocrystallites can be applied in fields of bone repair and regeneration. Therefore, the electrospun zein/HA fibrous membranes show promise for bone tissue engineering applications [117,118]. Zein also was associated with polyurethane/cellulose acetate (PU/CA), and electrospun nanofibrous scaffolds with diameters around 400 to 700 nm were prepared by physically blending zein and PU/CA [73]. The composite zein/PU/CA nano-scaffold showed an enhanced blood clotting ability in comparison with PU nanofibers. The presence of zein and CA in the nanofiber structure increased the hydrophilicity and bioactivity, and as consequence, a moist environment for the wound was formed, a fact that accelerated the wound recovery. The protein-based biomaterials possess excellent properties preferred for tissue engineering in relation to other types of structures. Wherefore, great efforts have been made to increase the water stability of electrospun protein scaffolds from cross-linking [73]. Jiang* et al.* [120] reported a new methodology of cross-linking electrospinning based on zein containing citric acid as a non-toxic cross-linker agent to improve the water stability and promote the good cytocompatibility property of zein fibers. In this case, electrospun zein fibers containing citric acid were obtained without using toxic catalysts. The good stability of these cross-linked fibers was evaluated in phosphate-buffered saline. The studies confirmed that the fibers promoted the proliferation of mouse fibroblast cells. The cross-linked electrospun fibers were stable in PBS after immersion for 15 days at 37 °C. Citric acid cross-linked electrospun zein scaffolds showed attachment and allowed the proliferation of fibroblast cells. On the other hand, zein fibers without citric acid did not promote fibroblast cell proliferation [120].

### 5.3. As Enzymatic Hydrolysate Peptides for Reducing Blood Pressure

Proline-rich proteins, for instance the γ-zein, present fragments, such as the Leu-Pro-Pro fragment, that inhibit the effect of the angiotensin-converting enzyme (ACE) and have applications in the medical field for lowering blood pressure. Angiotensin is a peptide hormone that promotes vasoconstriction and consequent augmentation of blood pressure. The ACE gene has the capability to produce instructions enabling angiotensin-converting enzyme. Maruyama* et al.* [121] obtained the sequence Val-His-Leu-Pro-Pro-Pro from γ-zein after hydrolysis using thermolysin and further ultrafiltration. In the same work, those authors synthesized such a sequence by the liquid phase method, using *N*,*N*-dimethylformamide as solvent with *N*,*N*-dicyclohexylcarbodiimide and 1-hydroxybenzotriazole as coupling reagents. They analyzed the potential of both (hydrolysate and synthetic) sequences for lowering the blood pressure in spontaneously hypertensive rats. However, due to strict reaction conditions, such a fragment is not easily produced from γ-zein by enzymatic hydrolysis. In this direction, Miyoshi* et al.* [122] demonstrated that α-zein can be hydrolyzed through various proteases and that the ultrafiltrated α-zein hydrolysates possess activity for reducing blood pressure. In addition, these authors demonstrated that when the unpurified thermolysin hydrolysate of α-zein was orally administered to spontaneously hypertensive rats, their blood pressure was reduced. Due to this, lots of works in this direction have been performed as reported in the review published by Garcia* et al.* [123]. Parris* et al.* [124] developed a dry- and wet-bioprocess to enhance the value of proteins from deoiled corn germ using different proteases. Most of the ACE-inhibitory peptides were in the <1 kDa fraction, after both wet- and dry-milled hydrolysates had been membrane fractionated. The control of total protein extracts (before treatment with proteases) from wet- and dry-milled germ showed that neither had ACE-inhibitory properties. Yamamoto* et al.* [125] provided a review in which biogenic peptides and their potential uses were described in terms of function, source and method of preparation of many peptides and also the mode of ACE inhibiting action. Furthermore, that review updated different works achieving strong blood pressure lowering activity for the peptide, Leu-Arg-Pro, isolated from α-zein hydrolysate prepared with thermolysin.

Ren* et al.* [126] demonstrated that sweeping frequency ultrasound (SFU) as a previous treatment of zein provided augmentation of the degree of hydrolysis (DH) in a solution (pH 8 using 1 M NaOH) at 50 °C containing alcalase enzyme (3500 U/g of protein). The authors verified that the SFU treatment not only increased the DH, but also, the hydrolysate presented a higher inhibitory effect on ACE. Ultrasonic pretreatment ruptures the fine meshwork structure of zein and causes the subsequent appearance of several micro-holes in the zein, as observed by scanning electron micrographs and atomic force micrographs. The results showed that sweeping frequency ultrasonic pretreatment promotes the release of ACE-inhibitory peptides from zein by altering the secondary structure and loosening the protein.

### 5.4. As Nutraceutical Zein Colloidal Particles

The development of colloidal particles for delivery systems, such as the encapsulation of micronutrients (vitamins, minerals and nutraceuticals), has increased recently. The incorporation of these kinds of micronutrients in food and beverage increases their functional behavior and has been considered as a way to improve human health through the diet [127]. Patel* et al.*’s research group has published since 2010 studies about the development and application of zein colloidal particles for nutraceutical encapsulation [128,129,130,131,132]. Patel* et al.* [128] prepared zein colloidal particles using sodium caseinate as an electrosteric stabilizer through the antisolvent precipitation method. They observed that the zein colloidal particles presented average size of 20 nm and a positively charged-surface. The addition of sodium caseinate in the antisolvent aqueous phase did not change the particle size, but change the surface charge that shifts from positive to negative. From FTIR analyses of the particles, they did not observe any chemical interaction between zein and sodium caseinate. The dispersion stability was studied in different pHs and electrolyte concentrations, from which sodium caseinate acted as a stabilizer of zein colloidal particles at neutral pH, and it was found that these particles had stability at high ionic strength and that the dried powder could be redispersed. According to the authors, “Because stabilization is merely due to the adsorption of protein on the particles, and the antisolvent used was water, this process is potentially easy to scale up. Such colloidal particles have the potential to be used as an all-natural biopolymer-based colloidal delivery system for encapsulating/embedding bioactive molecules in food (e.g., nutraceuticals), pharmaceutical (e.g., drug), and agricultural formulations”.

In another work, Patel* et al.* [129] studied the curcumin incorporation in zein colloidal particles, stabilized by sodium caseinate for oral delivery. It was verified that spherical zein:curcumin particles were obtained, presenting size around 100–150 nm. Moreover, the encapsulation efficiency decreased with the increase in curcumin proportion in the solution. Measurements of the photostability, pH stability and stability in simulated gastrointestinal conditions were carried and the authors observed that the pH and UV stabilities were improved with the curcumin encapsulation, mainly at physiological pH. Even more, the colloidal particles were stable in the simulated gastrointestinal conditions.

In 2012, Patel* et al.* [130] published an article about the encapsulation of quercetin, a polyphenol that presents physiological benefits to human health, such as antioxidant, anti-cancer and anti-viral activities [133]. The colloidal particles were prepared by precipitating simultaneously quercetin and zein into antisolvent, in the presence of sodium caseinate as an electrosteric stabilizer. The average size of the spherical-shaped particles ranged from 130 to 161 nm, and due to the presence of sodium caseinate, as the stabilizer, the particles’ surfaces became negatively charged, with the surface potential ranging from −30 to 41 mV. The antioxidant quercetin properties in the colloidal particles were studied by the ferric reducing anti-oxidant power (FRAP) method, which measures the antioxidant activity of quercetin for determining the stability of quercetin during photodegradation assays at different pHs. They observed that the antioxidant property of quercetin encapsulated in zein colloidal particles remained almost constant during the photodegradation study, as compared to quercetin in solution, indicating that encapsulation into zein colloidal particles enhanced quercetin’ molecular stability when exposed to UV irradiation at alkaline conditions.

In another work, Patel* et al.* [131] studied the encapsulation of curcumin (water insoluble) and indigo carmine (water soluble) in zein colloidal particles, used as colorants for yellow and blue, respectively. They verified that different shades of color in the yellow-green-blue range were generated by simply loading different ratios of curcumin and indigo carmine in the composite particles with an average particle size ranging from 76 to 300 nm. This information was presented in the recent review published by Patel and Velikov [132].

### 5.5. As Non-Conventional Biomaterial

Zein films with several microstructures such as channels and grids, were fabricated using the well-accepted soft lithography technique [134]. The micro features were successfully formed on zein matrices. The ease of fabrication at mild condition makes zein a potential candidate for being used as a microfluidic device base material. Leucha* et al.* [134] developed a green microfluidic device in which the manufacturing materials are made entirely of zein. The device can be utilized as disposable health and environmentally friendly microchips. From standard soft lithography and stereo lithography techniques, reliably fabricated thin zein films with diverse microfluidic designs were obtained [134]. Zein films with microfluidic channels were bonded on zein flat films and on glass slides by solvent bonding and vapor deposition methods. Once in contact with water, later on, the zein film becomes opaque due to protein precipitation. Therefore, a glass slide was used as another bonding surface for the sake of visualization. Therefore, zein can be a green alternative to non-degradable poly(dimethyl siloxane) (PDMS) and plastic material for microfluidic applications in agriculture [134]. Zein film can be bonded easily and quickly to different kinds of materials without requiring expensive equipment, such as an oxygen plasma generator. As a consideration for a more environmental-friendly and agricultural-based alternative to PDMS, zein microfluidic devices have been shown to have comparable bond strength and similar processes of fabrication that do not require any new equipment beyond standard ones. Zein microfluidic devices show distinct permeability to small molecules enabling diffusive exchange between fluid flows and bulk zein microfluidic channels. Zein microfluidic devices with micro mixing channels have the potential to be used as fluid manipulators that can be coupled with other analytical components for more complex micro total analysis applications. The flexibility and the ease of the bonding process of zein film make it a good candidate for fabrication into multilayer microfluidic devices. The main advantages of zein over PDMS is that zein, the renewable corn protein, has been demonstrated to be biodegradable, which is important for a “greener” approach in the field of portable and disposable microdevices. The application of zein microfluidic devices can be far reaching due to their biocompatibility, biodegradability and renewability [134]. The application of microfluidics in the agriculture sector is relatively new, and the number of research papers related to microfluidic applications in this field is growing rapidly, as is the interest in utilizing biodegradable materials, such as zein. Other biodegradable materials have been used as substrates for fabricating microfluidic devices, including silk fibroin and gelatin [134].

The control of the wettability of the surface and adsorption capacity of hydrophobic membranes is attractive for biomedical applications and electronic devices [135]. The most hydrophobic surfaces obtained from synthetic compounds are not biodegradable and still present low mechanical flexibility and are usually expensive. These factors reduce the potential applications of such materials. Therefore, biopolymers, such as zein, can make ease the obtainment of biomaterials with excellent properties, such as good surface wettability, good absorption potential, renewable, flexible, biodegradable and still inexpensive. Dong* et al.* [136] obtained, through a facile and inexpensive method, a zein based-material with a hydrophobic surface using self-assembly monolayer (SAM)-assisted evaporation-induced self-assembly (EISA).

## 6. Future Trends for Technological Applications of Zein-Based Materials

The biodegradability of zein is a key parameter for using this protein in several fields. New uses of zein and zein-based materials have grown rapidly in the last two decades as a result of recently developed technologies: (i) for extracting zein, mainly from corn and gluten, at lower costs; (ii) for mixing zein with other moieties (polymeric or not); (iii) for processing zein, allowing one to achieve different geometries and sizes, ranging from membranes, particles, films, fibers (nano- and micro-scale), ribbons,* etc.*; and (iv) for combining biodegradability and biocompatibility, making the usage of zein-blended materials in advanced technological fields possible, mainly as biomaterials (as a scaffold for tissue engineering, nutraceutical and drug carriers for controlled delivery,* etc.*), and also in conventional uses, such as food-packing and others.

It is important to stress that biomaterials possess a very high aggregated value. The fact that zein is extracted from renewable sources, at a gradually decreased costs, the potential for chemical modification of zein, due to the presence of different groups (amines, amides, hydroxyls, carboxylates and phenols) in the polymeric chain and the feasibility of processing (due to different developed plasticization ways), allowing different geometries, forms, sizes and hydrophilicity (depending of the other mixing components or derivatives), make zein a very important proteic material, with increasing demand.

However, some concerns still clearly remain as open opportunities for research targeting many more uses of zein, as follows:

(i) There are still few studies evaluating the influence of a chemical modifying process on the biodegradability of chemically-modified zein derivatives. Much of the works in the literature assume that the chemical modification of zein does not alter its biodegradability (or doing so at a very small scale, but neglected in deeper studies). In some cases, such an assumption might not be true;
(ii) The high biocompatibility (inexistence of toxicity) of neat zein can be affected after the plasticization process or by blending with other polymers. A quick search made in the ISI (Web of Science^©^) database using simultaneously the keywords “zein”, “plasticization” (or “blending”) and “cytotoxicity” revealed the inexistence of studies correlating the effects of plasticization (or blending) of zein to biocompatibility. Therefore, more cytotoxicity studies in this direction are needed.
(iii) The influence of the size and geometries on the biodegradability of zein and zein-based materials is an open issue. For instance, in the case of nanofibers or nanoparticles, the large exposed area may increase the rate of zein degradability, in an enzymatic environment.
(iv) According to a very recent publication [5], the authors pointed out zein-based nanocomposites with inorganic nanocrystal materials*.* The authors stated that these materials “…will undoubtedly draw more attention. These nanocomposites-based delivery systems are beginning to demonstrate their superior capability to encapsulate and control the release of drugs for a wide variety of applications…”. This statement is true. The incorporation of inorganic particles as encapsulated materials in zein-based nanocomposites (particles, nanofibers) opens the window for identifying/tuning different properties of zein and zein-based materials.

The above mentioned concerns are only some examples of the many other aspects that might be pointed out as future trends for zein and zein-based materials. The truth is that the both biodegradability and biocompatibility of zein and other inherent properties associated with this protein structure allow a myriad of applications with great potential in the near future.

## 7. Conclusions

This review describes and highlights some zein and zein-based materials, attempting to update the information concerning the basic structure, properties, changes of the properties (by chemical modification, blending, mixing), degradation (enzymatic or not) and applications of zein and zein-based materials, mainly in the food-packing, biomedical and pharmaceutical fields. It was demonstrated in this review, and in other works focused on zein and zein-based materials, that the biodegradation and biocompatibility of zein are key parameters for uses in biomedical, pharmaceutical and food-packing fields. Furthermore, it was pointed out that the hydrophilic-hydrophobic property of zein is a very important aspect for obtaining materials with different hydrophobicities to produce physical mixtures with other moiety (polymeric or not), but also for chemical reactions, obtaining derivatives with different properties.

The physical and chemical characteristics and special structure (at molecular, nano and micro scales) indicate that zein is inherently superior to many other polymers, natural or synthetic. As the delivery systems of zein-based materials are growing quickly, the applications will be expanded in a short time, mainly by developing advanced systems focused on zein-based micro- and nano-structures. There are already many nano- and micro-structured systems that have been developed from zein. Patel and Velikov [132] published a review in which the recent progress in the preparation of colloidal structures and their further application as functional materials were described. The film-forming property of zein and zein-based materials is important for the food and pharmaceutical industries. The good electrospinnability of zein is a good property that allows producing nanofibers of zein and zein-based materials mainly for applications in tissue engineering and controlled drug delivery. The use of zein’s hydrolysate peptides for reducing blood pressure is also a very important issue related to the application of zein’s derivatives in the biomedical field.

New studies focusing on the chemical modification of zein, the (bio)degradability and cytotoxicity of zein-based materials are still needed. The truth is that the biodegradability and biocompatibility of zein and other inherent properties associated with zein’s structure allow a myriad of applications of zein and zein-based materials with great potential in the near future.
